# Consortium of Lettuce and Beet in Succession to Green Manure Irrigated by Treated Dairy Effluent

**DOI:** 10.1002/wer.70221

**Published:** 2025-11-24

**Authors:** Juliana de Fátima Vizú, Rogers Ribeiro, Tamara Maria Gomes, Giovana Tommaso, Bruno Fernando Capodifoglio, Mileni Nobre Cabral, Ana Claudia Pereira Carvalho, Fabrício Rossi

**Affiliations:** ^1^ Luiz de Queiroz College of Agriculture University of São Paulo Piracicaba São Paulo Brazil; ^2^ Department of Foods Engineering, Faculty of Animal Science and Food Engineering University of São Paulo Pirassununga São Paulo Brazil; ^3^ Department of Biosystems Engineering, Faculty of Animal Science and Food Engineering University of São Paulo Pirassununga São Paulo Brazil

**Keywords:** agricultural reuse, *Beta vulgaris* L, dairy effluent, green manure, *Lactuca sativa* L, wastewater irrigation

## Abstract

Green manure as phytoremediation can help with the technical feasibility of growing vegetables irrigated with dairy effluent treated by an anaerobic system (ANE). The objective of this study was to evaluate the production of lettuce cultivated in conjunction with table beet following the irrigation of green manure with treated effluent from a dairy processing plant and its impact on the chemical characteristics of the soil. The experimental design utilized a randomized blocks, factorial scheme, employing two water sources (tap water [TW], dairy effluent treated by an ANE) and four green manures, with four replicates. At the 45‐day transplantation (DAT) mark, lettuce plants were analyzed, and at the 73‐day DAT, the table beet. Lettuce demonstrated enhanced productivity in succession to pigeon pea, irrespective of water source, with productivity values of 2.28 kg m^−2^ for TAP and 2.76 kg m^−2^ for ANE. The nutrient supply by the effluent had a positive influence on the production of table beet roots when in succession to jack bean, *Crotalaria juncea* , and pigeon pea, with values of 3.76, 3.50, and 3.50 kg m^−2^, respectively. Furthermore, the cultivation of lettuce and table beet in succession to green manures led to a reduction in sodium levels, resulting in a decrease in the exchangeable sodium percentage of irrigated soil treated with dairy effluent from 4.33% to 1.97%.

## Summary

- Effluent input positively influenced production of table beet roots when in succession of *Crotalaria juncea* , jack bean, and pigeon pea.
- Irrigation with dairy effluent treated by anaerobic system contributed to the chemical fertility of the soil.
- Green manure allows nutrients to be released and made available for crops in rotation or succession.
- Combining crops is a traditional practice for producing food and biomass, optimizing the use of natural resources.

## Graphical abstract

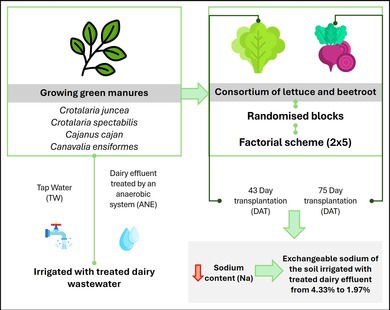

## 1 Introduction

Lettuce ( *Lactuca sativa* L.) is a leafy vegetable that is cultivated and consumed on a global scale. Despite its relatively low nutritional density in comparison to other vegetables (Chadwick et al. 2024; Ramírez‐Pedraza et al. 2024), lettuce consumption has been increasing, primarily due to its favorable flavor profile and high water content (95%), as well as its low caloric value, low fat content, and insignificant sodium values (Kathiravan et al. 2024; Shi et al. 2022). In Brazil, lettuce production exceeded 671,500 tons in 2021 (Kist and Beling 2023). Another vegetable that has commercial representativeness in Brazil and worldwide is table beet ( *Beta vulgaris* L.), which in 2016 corresponded to the equivalent average of 218,765 tons, with a planted area of 10,938 ha (ABCSEM 2017).

Vegetable production is an advantageous activity when carried out in environmental conditions and with a well‐developed market for commercialization. Consequently, there is a necessity to explore novel cultivation alternatives and technologies that facilitate enhanced productivity (Araújo et al. 2009).

In this regard, a practice that has gained traction and aligns with Sustainable Development Goal 6 (SDG 6: Sanitation and Drinking Water) is the reuse of treated wastewater for irrigation. This approach holds considerable promise in nourishing crops, conserving water resources, and reducing pressure on fresh water supplies (Mishra et al. 2023; Qing et al. 2021). However, it is important to note that there are also negative effects associated with the use of this treated wastewater. Primarily, it can contribute to the concentration of salts in the soil, which can lead to salinization and/or sodification (Pedrero and Alarcón 2009; Taylor et al. 2018).

Crops have a threshold level of tolerance to salinity, beyond which they begin to suffer salt stress and consequently lose production in proportion to the increase in salinity (Taiz and Zeiger 2013). Lettuce is considered moderately sensitive, whereas beetroot is moderately tolerant to salts.

Lettuce and beetroot intercropping can be used as a way of indirectly assessing the potential for use in salinized soils and/or sodified soils and/or phytoremediated soils, as well as the potential for use in saline soils (Ayers and Westcot 1999). Crop consortium is a traditional practice for producing food and plant biomass, and among the advantages offered by its adoption is the more effective use of natural resources, such as water and mineral nutrients (Humphries et al. 2004).

Another technique employed in sustainable production involves the incorporation of green manure into vegetable production systems, a practice that has garnered increased attention through various studies that have demonstrated its productive and economic effectiveness. This is primarily attributed to its capacity to release and make nutrients available to crops in rotation or succession (Kama et al. 2025; Dong et al. 2024; Persiani et al. 2024; Siwek et al. 2024; Shahrivar et al. 2023; Bento et al. 2015; Pauletti et al. 2009; Oliveira et al. 2008).

In this context, the objective of the present study was to evaluate the yield of lettuce cultivated in conjunction with table beet following the production of green manure irrigated with treated effluent from a dairy processing facility and its impact on the chemical composition of the soil.

## 2 Materials and Methods

### 2.1 Location and Experimental Design

The experiment was conducted in a protected environment, within an arched greenhouse with a total area of 210 m^2^, located at the Department of Biosystems Engineering, Faculty of Animal Science and Food Engineering (FZEA/USP) in the municipality of Pirassununga, São Paulo State, Brazil, at altitude of 627 m, latitude 21°59′S and longitude 47°25′W. The climate of the region is classified as Cwa according to the Köppen classification, that is, a subtropical climate with dry winters and hot summers (Köppen and Geiger 1928).

The experimental design used randomized blocks in a factorial scheme (2 × 5), totaling 10 treatments. The first factor was the water source (tap water [TW] and dairy effluent treated by an anaerobic system [ANE]), and the second factor consisted of four green manures, namely, *Crotalaria juncea* ( *crotalaria juncea* ), *Crotalaria spectabilis* ( *crotalaria spectabilis* ), *Cajanus cajan* (pigeon pea), and *Canavalia ensiformis* (jack bean), in addition to the control group (without green manure), with four replications, totaling 40 experimental plots.

The experimental plot was composed of a square fiberglass box, with a capacity of 0.5 m^3^ and surface area of 1 m^2^.

The green manures were sown on February 15, 2017, uniformly distributed in five rows per plot, at a depth of 2.0 cm. After thinning, 85, 90, 16, and 85 plants per plot were maintained for the green manures *C. juncea* , *C. spectabilis* , *C. ensiformis* , and *C. cajan* , respectively. The green manures were cultivated for 70 days after sowing (DAS). The plants located in the central area of the plot (0.5 m^2^) were cut and weighed to determine fresh biomass. A sample of the shoot biomass was placed in a paper bag and dried in a forced‐air circulation oven at 65°C for 72 h. After this period, the samples were weighed to determine dry mass. The material was subsequently ground and sent to the Laboratory of Soil Science, University of São Paulo (USP), for nutritional diagnostic analysis, following the methods described by Malavolta et al. (1997). After cultivating green manures, they were cut close to the ground, crushed, and left on the ground.

The curly lettuce seedlings “Vanda” and table beet “Cabernet” was transplanted on May 5, 2017. In the consortium, the plots were composed of two rows of table beet with nine plants each in spacing (0.25 × 0.10 m) and two rows of lettuce with four plants each in (0.25 × 0.25 m). The four central plants of lettuce and eight of table beet were considered as a useful plot.

### 2.2 Soil, Fertilization, and Climate

The soil for filling the boxes was classified as red latosol, medium texture, according to dos Santos et al. (2013). The chemical characterization of the soil, after the cultivation of green manures, was performed by samples composed of each experimental plot, in the 0.30 m layer, and analyzed at the Laboratory of Agricultural Sciences/ZAZ/FZEA, by the methodology described by Raij et al. (2001) (Table 1). The exchangeable sodium percentage was also determined (ESP), according to the equation: ESP = [(Na/CEC) * 100], in which ESP = exchangeable sodium percentage, Na = sodium (mmolc dm^−3^), and CEC = SB (Ca + Mg + K + Na) + H + Al, in mmolc dm^−3^.

**TABLE 1:** Description of the treatments.

Treatments	Water source	Green manure
T1	Tap water (TW)	*Crotalaria juncea*
T2	Tap water (TW)	*Crotalaria spectabilis*
T3	Tap water (TW)	*Cajanus cajan*
T4	Tap water (TW)	*Canavalia ensiformis*
T5	Tap water (TW)	*Control 1: without green manure*
T6	Dairy effluent treated by an anaerobic system (ANE)	*C. juncea*
T7	Dairy effluent treated by an anaerobic system (ANE)	*C. spectabilis*
T8	Dairy effluent treated by an anaerobic system (ANE)	*C. cajan*
T9	Dairy effluent treated by an anaerobic system (ANE)	*C. ensiformis*
T10	Dairy effluent treated by an anaerobic system (ANE)	*Control 2: without green manure*

The same procedures and analyses were performed for the soil after harvesting the lettuce and table beet plants, in addition to making saturation paste using the methodology of Richards (1954), to determine the electrical conductivity and pH of the saturation extract.

The recommendation for planting fertilization was carried out according to the result of the soil analysis (Table 2), which did not justify different fertilization in plots previously irrigated with the two sources of water (TW and ANE). According to the needs of the cultures (Raij et al. 1997), two were applied: 20 t ha^−1^ of organic compost (1.7% of N, 1.24% of K_2_O, 14.45% of Ca, 0.7% of Mg 0.74% of S, and micronutrients) and 300 kg ha^−1^ of P_2_O_5_, in the form of magnesian thermophosphate (Yoorin Master 1 Si), which provides in addition to phosphorus, calcium (18% Ca), magnesium (7% Mg), boron (0.10% B), copper (0.05% Cu), manganese (0.30% Mn), silicon (10% Si), and zinc (0.55% Zn).

**TABLE 2:** Result of the chemical analysis of the soil after the cultivation of green manures irrigated with tap water (TW) or dairy effluent treated by anaerobic system (ANE), in the layer of 0–30 cm.

Treatments	pH (CaCl_2_)	P	S	K	Ca	Mg	Na
		mg dm^−3^		mmolc dm^−3^			
TW	6.50b	27.45b	8.80a	2.49a	54.20 b	13.60b	0.28b
ANE	6.55a	30.60a	8.95a	2.60a	57.25 a	18.10a	4.20a
CV (%)	1.44	17.74	38.63	12.57	5.90	13.97	26.49

Treatments	OM	H + Al	SB	CEC	V	ESP
	g kg^−1^	mmol_c_ dm^−3^			(%)	(%)
TW	17.19a	14.51a	70.57 b	85.10 b	82.86 b	0.33 b
ANE	17.88a	13.92b	82.15 a	96.05 a	85.47 a	4.39 a
CV (%)	9.06	3.55	6.49	5.34	1.34	28.64

*Note:* Averages followed by different letters, for each parameter, differ from each other by the test F (*p* < 0.05).

It was also added 150 kg ha^−1^ of K_2_O in the form of boiler ash, obtained from the boiler that supplies the dairy itself. During the cultivation period of the consortium, there was also the decomposition of green manures. Only in the plots irrigated with TW there was a covering fertilization with 15 kg ha^−1^ of nitrogen (N), in the form of ammonium nitrate.

The maximum relative humidity throughout the consortium period was 81%, and the minimum relative humidity was 20%, whereas the maximum temperature was 34.3°C, and the minimum was 12.7°C. Suitable temperatures for lettuce are between 15°C and 20°C and for table beet between 10°C and 20°C (Trani et al. 2014). Crop development was adequate in the climatic conditions of the agricultural greenhouse.

### 2.3 Water Sources

The effluent used in the irrigation of the experiment came from the Dairy School on the USP campus “Fernando Costa,” Pirassununga, and TW was diverted from the pipe that supplied the greenhouse. The effluent treatment was carried out by ANE, with immobilized biomass and batch and hydraulic detention time of 48 h.

The physical–chemical characterization of the treated effluent and TW was carried out weekly, with the samples being collected according to the National Guide for Collecting and Preservation of Water Samples (CETESB/ANA 2011) and analyzed according to APHA, AWWA, and WEF (2012). The parameters analyzed were electrical conductivity (EC), pH, nitrogen series (NTK, NH_4_^+^, NO_3_^−^, and NO_2_^−^), total and soluble phosphorus (P), potassium (K), sulfate (SO4^−^), chlorine (Cl), iron (Fe), manganese (Mn), calcium (Ca), magnesium (Mg), and sodium (Na); the last three were used to determine the sodium adsorption ratio (SAR) using the equation described by Ayers and Westcot (1999).

The posttreatment effluent by anaerobic reactor was filtered in a geotextile cover to remove coarse solids and later disinfected by ultraviolet radiation to remove pathogenic microorganisms.

### 2.4 Irrigation

The irrigation system adopted was superficial drip, with integrated and non‐compensating drippers, spaced every 0.20 m, with a flow rate of 2.3 L h^−1^ and a service pressure of 15 m.c.a., through which different water sources (ANE and TW) were applied for irrigation of intercropped cultivation.

The system consisted of automatically activated pumping systems, hydrometers, and glycerin gauges to control the flow and pressure, respectively, in addition to a 120‐mesh disc filter, for removing suspended solids.

Irrigation management was based on the replacement of the crop evapotranspiration estimate (ETc), calculated by the evaporation of the reduced class A tank, corrected by the average crop coefficient (Kc), as proposed by the Food and Agriculture Organization of the United Nations (FAO 2008) at the different stages of crop development. The reduced tank had a diameter of 0.60 and 0.25 m in height and was installed in the central part of the greenhouse. The tank correction coefficient (Kp) used for the reduced condition, in a protected environment, was 1, as mentioned by Farias et al. (1994). The 2‐day watering shift was adopted.

**FIGURE 1:**
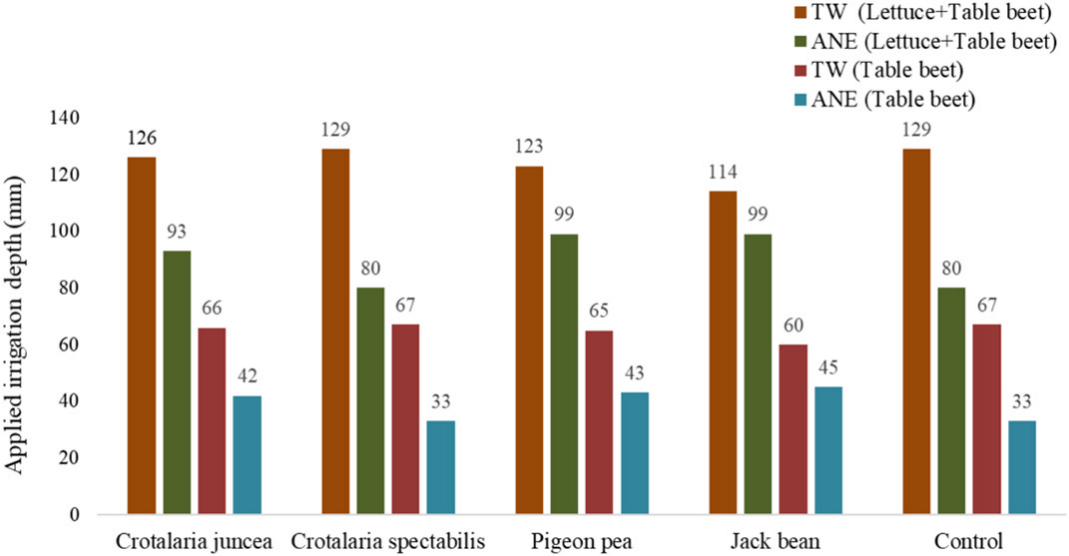
Irrigation depths (mm) of tap water (TW) and dairy effluent treated by an anaerobic system (ETL) applied to the treatments during the lettuce and beet intercropping period and after lettuce harvest on the beet plants.

The irrigation frequency adopted was daily, and the actual volume applied during irrigation was recorded using water meters. The applied volumes during the intercropping period of lettuce and beet, as well as from lettuce harvest until beet harvest, are presented in (Figure 1).

### 2.5 Consortium Cultivation

Lettuce and table beet plants were grown, respectively, for 45 and 73 days after transplantation (DAT). The four lettuce plants from the central area of the plot and eight table beet plants were cut close to the soil surface and separated into leaves and roots. After separation, they were weighed on an analytical balance to determine the fresh mass of the shoot and roots.

A sample of the aerial part and roots were packed in paper bags and dried in an oven at 65°C for 72 h, and the dry mass was subsequently determined.

The height of the plants was determined with the aid of a ruler, from the base of the plant to the maximum height of the leaves. To determine the number of leaves and leaf area, only leaves with a height equal to or greater than 0.05 m were considered.

The leaf area was measured by a leaf area integrator, brand Li‐Cor, model LI‐3100C. The diameters of the aerial part of the lettuce plants and the roots of the table beet were also determined. Three table beet roots from each plot were crushed, their juice was extracted and the total soluble solids (TSS) (^o^Brix) were determined by a digital refractometer, brand Reichert.

Samples of the dry leaves of lettuce and table beet were sent to the Laboratory of Agricultural Sciences/ZAZ/FZEA for leaf analysis according to the methodology proposed by Malavolta et al. (1997).

### 2.6 Analysis of Results

The results of the physical–chemical characterization of effluents and TW were presented by mean values and standard deviation. Statistical analyses of soil and plant results were performed according to the randomized block design, with analysis of variance (ANOVA) being performed, and the means compared by the Tukey or *F* test at 5% probability, using the statistical program SISVAR 5.6 (Ferreira 2011).

## 3 Results

### 3.1 Water Sources

The characterization of the effluent treated by ANE presented considerable amounts of nitrogen (N‐TNK = 70.92 ± 35.80 mg L^−1^), potassium (K = 74.80 ± 50.84 mg L^−1^), magnesium (Mg = 69.47 ± 43.49 mg L^−1^), and calcium (Ca = 40.89 ± 35.81 mg L^−1^), higher than TW (Table 3). Gomes et al. (2017) studying the supplementation of nutrients for table beet using anaerobic effluent reported the concentration of N‐TNK = 42.24 ± 1.77, lower than this study.

**TABLE 3:** Physicochemical characterization of water sources used for irrigation of intercropped cultivation of lettuce and table beet.

Parameter	Dairy effluent treated by anaerobic system (ANE)	Tap water (TW)
N‐TNK (mg L^−1^)	70.92 ± 35.80	19.46 ± 2.47
N‐NH_4_^+^ (mg L^−1^)	36.81 ± 27.82	0.00 ± 0.00
N‐NO_3_^−^ (mg L^−1^)	0.07 ± 0.08	0.02 ± 0.05
N‐NO_2_^−^ (mg L^−1^)	0.58 ± 0.28	0.00 ± 0.00
P Total (mg L^−1^)	5.63 ± 1.61	0.21 ± 0.17
P Solúvel (mg L^−1^)	1.78 ± 0.94	0.05 ± 0.03
K^+^ (mg L^−1^)	74.80 ± 50.84	0.23 ± 0.05
Ca^+2^ (mg L^−1^)	40.89 ± 35.81	6.85 ± 1.06
Mg^+2^ (mg L^−1^)	69.47 ± 43.49	1.83 ± 0.27
Na^+^ (mg L^−1^)	197.20 ± 101.01	1.78 ± 0.65
Cl^−^ (mg L^−1^)	160.52 ± 67.9	2.39 ± 1.98
Fe^++^ (mg L^−1^)	0.03 ± 0.01	0.15 ± 0.00
Mn^++^ (mg L^−1^)	0.04 ± 0.04	0.01 ± 0.01
EC (dS m^−1^)	2.74 ± 0.76	0.04 ± 0.02
pH	7.77 ± 0.41	6.92 ± 0.18
SAR (mmol L^−1^)^‐1/2^	4.35 ± 1.68	0.16 ± 0.05

Abbreviations: EC = electric conductivity, SAR = sodium adsorption ratio, TNK = total nitrogen Kjeldahl.

The saline potential of the source with dairy effluent is observed by the values Cl, Na+, EC, and SAR (Table 3). According to the guidance of CETESB (2006), the maximum concentrations of sodium and chlorine to prevent sodification and/or salinization of the soil are 69 and 106 mg L^−1^, respectively, for reuse of domestic treated wastewater in agriculture. Thus, the values found are above those recommended by the environmental agency, indicating that there is a saline potential of this effluent for application to the soil. However, the values of EC and SAR are within the acceptable range, a maximum value of 12 for SAR and 2.9 dS m^−1^ for EC (CETESB 2006) Despite this, it is important to note that the average EC of the treated dairy effluent was above the tolerable range by lettuce (1.3 dS m^−1^), and bordering on table beet (2.7 dS m^−1^), to guarantee 100% of the crop's productive potential (Ayers and Westcot 1999).

### 3.2 Green Manure Biomass Production and Nutrient Concentrations

The shoot fresh biomass (SFB) of jack bean ( *C. ensiformis* ) showed no significant difference in productivity between water sources, indicating that jack bean is suitable for irrigation with dairy effluent treated by an ANE. The other green manures, however, produced lower SFB when irrigated with ANE compared to TW (tap water) (Table 4).

**TABLE 4:** Fresh and dry shoot biomass of green manures under tap water (TW) and anaerobically treated dairy effluent (ANE) irrigation.

Treatments	TW	ANE	Mean
	Shoot fresh biomass (SFB) t ha^−1^		
Jack bean	19.3Aa	18.5Aa	18.8
*Crotalaria spectabilis*	19.9Aa	11.1Bb	15.5
*Crotalaria juncea*	12.0Ba	8.4Cb	10.2
Pigeon pea	8.1Ca	4.9Db	6.5
Mean	14.8	10.7	
CV (%) = 9.87			
	**Shoot dry biomass (SDB)** **t ha** ^**−1**^		
Jack bean	5.1	4.6	4.9A
*C. spectabilis*	3.2	2.3	2.8B
*C. juncea*	3.7	2.1	2.9B
Pigeon pea	2.5	1.5	2.0C
Mean	3.6a	2.6b	
CV (%) = 13.8			

*Note:* Means followed by different letters, lowercase in rows and uppercase in columns, differ from each other according to the *F* test and Tukey's test (*p* ≤ 0.5), respectively.

Abbreviation: CV = coefficient of variation.

Analyzing the green manures within each water source, jack bean ( *C. ensiformis* ) showed the highest SFB when irrigated with ANE, followed by *C. spectabilis* , *C. juncea* , and dwarf pigeon pea ( *C. cajan* ). Regarding tap water (TW), jack bean and *C. spectabilis* exhibited the highest SFB, differing from *C. juncea* and dwarf pigeon pea, which showed the lowest productivity.

Overall, the SFB productivity obtained for jack bean in this experiment, regardless of the water source, exceeds the values reported by Cavalcante et al. (2012), who obtained 16.2 and 13 t ha^−1^ in their field studies, respectively, with harvests carried out after 92 and 119 days of cultivation. The results for *C. spectabilis* (39.10 t ha^−1^) and dwarf pigeon pea (25 t ha^−1^) were lower than those reported by Pereira et al. (2012). For *crotalaria juncea* , the values were below those cited by Cavalcante et al. (2012), and the lower fresh biomass productivity of these species may possibly be explained by their higher sensitivity to irrigation with anaerobically treated dairy effluent, which, combined with powdery mildew (*Oidium* sp.) infection, caused severe defoliation in *C. juncea* .

The results for shoot dry biomass (SDB) productivity showed that all green manures exhibited higher productivity when irrigated with TW, with jack bean showing the highest productivity (5.1 t ha^−1^) and dwarf pigeon pea the lowest (2.5 t ha^−1^), whereas *crotalaria spectabilis* and *crotalaria juncea* did not differ from each other. The means for *crotalaria spectabilis* and *crotalaria juncea* were also lower when compared with the results obtained by Soares et al. (2015), who found 4.2 and 10.49 t ha^−1^, respectively. It is worth noting that the cited references refer to field cultivation conditions. In a study similar to the present one, Rossi et al. (2014) reported higher SDB values for pearl millet plants irrigated with dairy effluent treated by an anaerobic system, with mean values of 3.8 t ha^−1^.

No interaction was observed between water sources and green manures for nitrogen (N) and sulfur (S) concentrations, and higher phytoextraction of these macronutrients was observed under TW irrigation (Table 5).

**TABLE 5:** Macronutrient phytoextraction by the biomass of green manures irrigated with tap water and dairy effluent treated by an anaerobic system.

Treatments	TW	ANE	Mean
N (kg ha^−1^)			
Jack bean	134.1	112.8	123.5A
*Crotalaria spectabilis*	88.9	79.0	84.0B
*Crotalaria juncea*	82.2	60.1	71.2B
Pigeon pea	48.3	38.5	43.4C
Mean	88.4a	72.6b	
CV (%) = 21.62			
P (kg ha^−1^)			
Jack bean	2.8	3.8	3.3A
*C. spectabilis*	2.2	2.6	2.4B
*C. juncea*	2.0	1.8	1.9B
Pigeon pea	1.5	1.6	1.6B
Mean	2.2a	2.5a	
CV (%) = 26,36			
K (kg ha^−1^)			
Jack bean	63.5	68.1	65.8A
*C. spectabilis*	70.4	53.8	62.1A
*C. juncea*	42.1	40.3	41.2B
Pigeon pea	28.4	23.6	26.0C
Mean	51.1a	46.4a	
CV (%) = 17.34			
Ca (kg ha^−1^)			
Jack bean	86.6Aa	73.3Ab	80.0
*C. spectabilis*	60.9Ba	33.7Bb	47.3
*C. juncea*	15.0Ca	9.1Cb	12.0
Pigeon pea	13.4Ca	8.6Cb	11.0
Mean	44.0	31.2	
CV (%) = 14.89			
Mg (kgha^−1^)			
Jack bean	5.0Ba	6.7Aa	5.8
*C. spectabilis*	14.0Aa	8.6Ab	11.3
*C. juncea*	4.1BCb	6.5Aa	5.3
Pigeon pea	1.7Ca	1.6Ba	1.6
Mean	6.2	5.9	
CV (%) = 23.54			
S (kg ha^−1^)			
Jack bean	9.9	8.9	9.4A
*C. spectabilis*	7.1	4.4	5.7B
*C. juncea*	6.0	4.9	5.5B
Pigeon pea	4.7	2.8	3.7C
Mean	6.9a	5.3b	
CV (%) = 13.67			
Na (kg ha^−1^)			
Jack bean	5.1Aa	4.7Aa	4.8
*C. spectabilis*	3.7Ba	4.2Aa	3.9
*C. juncea*	3.2Bb	4.6Aa	3.9
Pigeon pea	2.5Ba	1.5Bb	2.0
Mean	3.6	3.8	
CV (%) = 17.02			

*Note:* Means followed by different letters, uppercase in columns and lowercase in rows, differ from each other according to Tukey's test and the *F* test (*p* ≤ 0.5), respectively.

Jack bean showed the highest mean nitrogen (N) content (123.5 kg ha^−1^), followed by *crotalaria spectabilis* (84.0 kg ha^−1^) and *crotalaria juncea* (71.2 kg ha^−1^), which did not differ from each other, and dwarf pigeon pea, which presented the lowest mean value (43.4 kg ha^−1^). A similar trend was observed for sulfur (S). The higher N accumulation in jack bean compared to the other green manures is related to its greater biomass productivity. Cavalcante et al. (2012) reported N phytoextraction values of 79.5 kg ha^−1^ for *crotalaria spectabilis* , 65 kg ha^−1^ for *crotalaria juncea* , 71 kg ha^−1^ for jack bean, and 107.2 kg ha^−1^ for dwarf pigeon pea.

Interactions were observed between the factors water sources and green manures for both calcium (Ca) and magnesium (Mg). For calcium, a consistent reduction was observed when plants were irrigated with anaerobically treated dairy effluent (ANE) compared to TW. Jack bean stood out as the species with the highest phytoextraction under both water sources. These results indicate that ANE reduced the availability and uptake of Ca by plants, possibly due to the higher sodium content in the effluent, which may promote ionic competition and displacement of Ca^2+^ from soil exchange sites, thus impairing the root absorption of this nutrient.

For magnesium, distinct responses were found among the green manure species. Significant differences in Mg levels were observed only for pigeon pea when ANE was used as the irrigation source, with mean values of 8.6 kg ha^−1^ for *crotalaria spectabilis* , 6.7 kg ha^−1^ for jack bean, 6.5 kg ha^−1^ for *crotalaria juncea* , and 1.6 kg ha^−1^ for pigeon pea. Under TW irrigation, the highest Mg phytoextraction was recorded for *C. spectabilis* (14.0 kg ha^−1^), followed by jack bean (5.0 kg ha^−1^), which did not differ from *C. juncea* (4.1 kg ha^−1^) but was superior to pigeon pea (1.7 kg ha^−1^). *C. spectabilis* exhibited a significant reduction in Mg uptake (38.6%) when irrigated with ANE. Conversely, *C. juncea* showed the opposite trend, with a marked increase (58.5%) in Mg phytoextraction under ANE irrigation, whereas jack bean and pigeon pea did not show significant differences between water sources. This response suggests that the use of ANE is influenced by physiological and adaptive traits specific to each species, reflecting differences in their capacity to absorb and transport bivalent cations under higher salinity conditions.

Garcia et al. (2007) observed that irrigation with saline water reduced the uptake of K, Ca, and Mg by maize plants. Similarly, dos Santos et al. (2017) reported that increasing salinity in nutrient solutions prepared with brackish water led to reductions in potassium and nitrogen concentrations in cherry tomato leaf tissue.

The phytoextraction of sodium (Na) by green manure species showed a significant interaction between factors, revealing distinct behaviors among species in response to irrigation with ANE and TW. In general, irrigation with ANE increased Na uptake compared with TW, except for pigeon pea. The water source did not affect Na phytoextraction in jack bean, which showed similar values under TW (5.1 kg ha^−1^) and ANE (4.7 kg ha^−1^), nor in *C. spectabilis* (3.7 kg ha^−1^ under TW and 4.2 kg ha^−1^ under ANE), indicating tolerance to sodium and stability in Na absorption. Pigeon pea exhibited the lowest mean Na uptake (1.5 kg ha^−1^ under ANE), differing significantly from the other species, which demonstrates its lower capacity to absorb and accumulate sodium in the shoot. Under TW, jack bean presented the highest mean value, differing from the other treatments.

The overall mean Na phytoextraction increased slightly under ANE irrigation (3.8 kg ha^−1^) compared with TW (3.6 kg ha^−1^), supporting the effect of the higher Na concentration in the effluent (197 ± 101.1 mg L^−1^ vs. 1.78 ± 0.65 mg L^−1^ in TW). These findings indicate that although the anaerobically treated effluent increases sodium input into the system, green manure species exhibit limited phytoextraction capacity, insufficient to prevent Na accumulation in the soil. This limitation may contribute to increases in ESP and SAR, as previously reported by Rossi et al. (2014) and Donatti et al. (2017), who found higher Na accumulation in soils irrigated with effluents due to the low removal of this element by plant biomass.

### 3.3 Lettuce Productivity and Biometric Aspects

The analysis for fresh mass of the aerial part of the lettuce (FMAP) and fresh mass of the leaves (FML) showed an interaction among water sources and green manures. Lettuces grown in succession to pigeon pea obtained the highest averages regardless of the source of water received during irrigation (Table 6).

**TABLE 6:** Fresh mass from the aerial part (FMAP), fresh mass from the leaves (FML), number of leaves (NL), plant diameter (DIA), plant height (PHG), and leaf area index (LAI) of lettuces irrigated with tap water (TW) and dairy effluent treated by anaerobic system (ANE).

Treatments	FMAP		FML		NL	
	kg m^−2^		kg m^−2^		Leaves plant^−1^	
	TW	ANE	TW	ANE	TW	ANE
Pigeon pea	2.28Aa	2.76Aa	1.92Aa	1.84Aa	16.7Aa	16.2Aa
*Crotalaria juncea*	2.04ABa	2.24Aa	1.58Aa	1.94Aa	15.6Aa	16.1Aa
Jack bean	1.68ABb	2.44Aa	1.40Ab	2.22Aa	15.0Aa	17.4Aa
*Crotalaria spectabilis*	1.94ABa	2.42Aa	1.50Aa	1.80Aa	15.5Aa	15.6Aa
Control	1.32Ba	0.78Ba	1.08Aa	0.58Ba	14.6Aa	10.6Bb
CV (%)	24.52		27.98		11.61	

Treatments	DIA		PHG		LAI	
	cm plant^−1^		cm plant^−1^		m^2^ m^−2^	
	TW	ANE	TW	ANE	TW	ANE
Pigeon pea	24.48Aa	24.72Aa	23.3Aa	23.5Aa	4.12Aa	3.90Aa
*C. juncea*	22.67ABa	24.28Aa	21.7ABa	23.0Aa	3.66Aa	4.14Aa
Jack bean	21.50ABb	24.93Aa	21.5ABb	24.9Aa	2.94Ab	4.87Aa
*C. spectabilis*	22.13ABa	23.08Aa	23.3Aa	23.7Aa	3.60Aa	4.21Aa
Control	19.63Ba	16.96Bb	18.9Ba	17.3Ba	2.78Aa	1.59Ba
CV (%)	6.98		9.02		25.56	

*Note:* Means followed by different letters, uppercase in the columns and lowercase in the rows, differ from each other by the Tukey and *F* test (*p* < 0.5), respectively.

Abbreviation: CV = coefficient of variation.

Lettuces grown in succession to pigeon pea, irrigated with TW had the highest average (FMAP = 2.28 kg m^−2^), differing from the control. The same behavior was observed in relation to ANE, and the highest averages were for pigeon pea 2.76 kg m^−2^ and the lowest mean for control 0.78 kg m^−2^. Purquerio et al. (2011) obtained higher productivity of fresh mass of lettuce cv. Camila, conducted for 38 days, after the cultivation of millet and *C. juncea* in salinized soil than the control (without cover plants) of 2.26 and 2.50 kg m^−2^, respectively. Oliveira et al. (2008) observed that the fresh mass of the lettuce was higher when legumes were used as ground mulch, due to nitrogen fixation. Sandri et al. (2007) stated that lettuce productivity can be higher in irrigation systems by underground and superficial drip using wastewater. For fresh leaf mass (FML), when the water source was TW, the highest average was for pigeon pea (1.92 kg m^−2^) and the lowest average for control (1.08 kg m^−2^). Jack bean had its average influenced by the water source, producing less FML when irrigated with TW (1.40 kg m^−2^) in relation to ANE, and this treatment presented the highest productivity (2.20 kg m^−2^), and for the same source of water, the control showed the lowest values of 0.58 kg m^−2^. These values were higher for all treatments, except for the control when irrigated with TW, to those found by Sandri et al. (2007) who found values of 0.84 kg m^−2^ for FML in plants irrigated with treated domestic effluents, cultivated for 45 days. However, it is necessary to consider that in addition to the nutrients provided by the ANE, there is the decomposition of the green manure cover.

The fresh (FRM) and dry root (DRM) mass of the lettuce showed no statistical differences in relation to the treatments. The average value for FRM when the plants were irrigated with TW was 0.26 and 0.22 kg m^−2^ for ANE. For DRM, the averages were: 0.134 kg m^−2^ for TW and 0.124 kg m^−2^ for ANE.

The results obtained for diameter (DIA) showed that the lettuce plants did not show differences in size when in successive cultivation to the pigeon pea, regardless of the water source (Table 3). Lettuces grown in succession to pigeon pea showed the highest averages for DIA when irrigated with TW, not differing from the other treatments, only from the control. When irrigated with ANE, lettuces in successive pigeon pea cultivation showed the highest average (24.72 cm), differing only from the control (16.96 cm). Juchen et al. (2013) found DM values for lettuce plants irrigated with 33.12 cm dairy wastewater after 55 days of cultivation.

When plant height (PHG) was evaluated, it was observed that lettuces irrigated with TW in cultivation after *C. spectabilis* and pigeon pea had the highest averages, followed by *C. juncea* and jack bean, which did not differ from the control (Table 3). In cultivation irrigated with ANE, lettuces in succession to jack bean showed the highest mean for TW (24.93 cm), differing only from the control with the lowest mean, 17.25 cm. Juchen et al. (2013) found height values for lettuce plants irrigated with dairy waste water of 21.06 cm, similar to the results found by Sandri et al. (2007), who found an average of 22.1 cm.

Despite the differences found for fresh leaf weight (FML), the number of leaves (NL) showed a difference only for the control when irrigated with ANE, which had the lowest amount (10.6 leaves). Magalhães et al. (2015) found the following averages for number of leaves: 10.75 leaves cv. Rapids, 10.38 leaves cv. Monica, and 13.63 leaves for cv. Simpson.

The leaf area index (LAI) showed interaction among treatments, for plants irrigated with TW the highest averages were observed by lettuce in succession to pigeon pea cultivation; however, it did not differ from the other treatments. When the plants were irrigated with ANE, the highest averages were for jack bean, which differed from the control. It is possible to observe that the lettuce in succession to jack bean had a higher LAI when it received TW. The fact that lettuces in succession to pigeon pea, when irrigated with TW, had higher LAI is related to their greater production of fresh mass, total number of leaves, and height, as well as lettuce plants in post‐cultivation to jack bean when irrigated with ANE.

In general, the fact that lettuce plants in successive cultivation to green manures have performed well when irrigated with ANE may be related to the supply of nutrients provided by the effluent together with the release of nutrients by the decomposition of green manures in the system.

### 3.4 Table Beet Productivity and Biometric Aspects

There was interaction for the successive cultivation in a combination of lettuce and table beet post‐cultivation of green manures and water sources for total fresh mass (TFM), FML, number of leaves (NL), fresh root mass (FRM), and diameter (DIA) of the table beet plants (Table 4). The leaf area index showed a difference only for water sources.

The beets, after the cultivation of *C. spectabilis* , and the control were not affected, regardless of the source of water used for TFM, and for the beets in succession with other green fertilizers, the highest TFM was when irrigated with dairy effluent treated by anaerobic system (ANE).

The highest production of fresh mass from the leaves (FML) irrigated with TW occurred in the control treatments and with *C. spectabilis* coverage, followed by the pigeon pea, *C. juncea* , and jack bean (Table 4). Sediyama et al. (2011) found greater production of FML for table beet plants when cultivated under mulch. For plants irrigated with ANE, the highest averages were for the pigeon pea, jack bean, *C. juncea* , control, and *C. spectabilis* (Table 7). When assessing the fresh mass of table beet leaves irrigated with different sources of water and irrigation blades, Gomes et al. (2015) found in the irrigation blades 100% replacement of the evapotranspiration of the culture and with anaerobic effluent treated with dairy a fresh leaf mass of 1.28 kg m^−2^.

**TABLE 7:** Total fresh mass (TFM), fresh leaf weight (FML), number of leaves (NL), root diameter (DIA), fresh root weight (FRM), and leaf area index (LAI) of plants of table beet irrigated with tap water (TW) and dairy effluent treated by anaerobic system (ANE).

Treatments	TFM		FML		NL	
	kg m^−2^		kg m^−2^		Leaves plant^−1^	
	TW	ANE	TW	ANE	TW	ANE
Pigeon pea	2.94Ab	5.92Aa	0.82Ab	2.42Aa	5.94Ab	9.31ABa
*Crotalaria juncea*	2.90Ab	5.60Aa	0.82Ab	2.08ABa	5.92Ab	9.82Aa
Jack bean	1.74Ab	5.98Aa	0.56Ab	2.22ABa	5.25Ab	8.34ABCa
*Crotalaria spectabilis*	3.40Aa	3.96Aa	0.92Aa	1.32Ba	6.31Aa	6.42Ba
Control	3.58Aa	4.74Aa	0.94Aa	1.42Ba	6.40Aa	6.87BCa
CV (%)	28.69		32.27		30.13	

Treatments	FRM		DIA		LAI^n.s.^	
	kg m^−2^		cm plant^−1^		m^2^ m^−2^	
	TW	ANE	TW	ANE	TW	ANE
Pigeon pea	2.12Ab	3.50Aa	4.22ABa	4.91Aa	1.41	3.18
*C. juncea*	2.06Ab	3.50Aa	4.09ABb	4.97Aa	1.20	2.99
Jack bean	1.16Ab	3.76Aa	3.38Bb	5.16Aa	3.38	5.16
*C. spectabilis*	2.46Aa	2.62Aa	4.48Aa	4.60Aa	1.36	2.13
Control	2.62Aa	3.30Aa	4.22ABa	4.94Aa	1.58	2.18
CV (%)	29.86		11.52		16.99	

*Note:* Means followed by different letters, uppercase in the columns and lowercase in the rows, differ from each other by the Tukey and *F* test (*p* < 0.5), respectively.

Abbreviations: CV = coefficient of variation, n.s. = not significant.

Regarding the number of leaves (NL), the beets irrigated with TW showed no differences in relation to green manures. When the beets were irrigated with ANE, the highest average NL was for table beet in succession to *C. juncea* (9.82 leaves plant^−1^), differing from table beet after *C. spectabilis* (6.82 leaves plant^−1^). Note that for the jack bean, pigeon pea, and *C. juncea* treatments, irrigation with treated effluent (ANE) contributed to the largest amount of leaves. Silva et al. (2013), when studying the performance of table beet cultivars under different water tensions in the soil, found average values of 9.96 leaves plant^−1^ for the Early Wonder cultivar and 10.20 leaves plant^−1^ for the cultivar Itapuã. Sarmento et al. (2011) found a value of 10.28 leaves plant^−1^ for table beet grown with organic fertilization.

In the interaction among treatments for FRM, the beets irrigated with TW had the highest averages for cultivation without green manure (control) (2.62 kg m^−2^) and *C. spectabilis* (2.46 kg m^−2^), which differed from the beets after jack bean (1.16 kg m^−2^). Regarding the plants irrigated with ANE, no difference was observed, with the highest average being for the table beet in succession to jack bean (3.76 kg m^−2^) and the lowest after *C. spectabilis* (2.62 kg m^−2^). In this study, it can be seen that the roots had a better development in irrigation by ANE in succession to jack bean (3.76 kg m^−2^), pigeon pea (3.50 kg m^−2^), and *C. juncea* (3.50 kg m^−2^). These values were similar to those found by Gomes et al. (2015) show in their studies an average of 3.18 kg m^−2^.

The treatments that received ANE were efficient in the production of table beet, because they meet the market preference, which, according to Sediyama et al. (2010), the preferred root mass is between 200 and 300 g.

Regarding the dry mass of the table beet roots, the difference occurred in relation to water sources, in TW the average production was 0.48 kg m^−2^, whereas for ANE the average was 0.72 kg m^−2^. Gomes et al. (2015) also found the highest mean for dry mass of the roots when the beets were irrigated with treated dairy effluent in relation to TW.

The highest mean for root diameter (DIA), in relation to irrigation with TW, was for beets cultivated in succession to *C. spectabilis* (4.48 cm), which differed from beets after jack bean (3.38 cm). In irrigation with TW, the DIA of the beets did not show statistical differences, the highest value being obtained with the beets in succession to jack bean (5.16 cm). Gomes et al. (2015) found higher values, DIA = 6.5 cm, for table beet plants irrigated with dairy treated effluent and cultivated for a period of 72 days. Silva et al. (2017) that in post‐cultivation of cover, plants present DR values of 5.92 cm after *Crotalaria* cultivation and 6.78 cm for plants cultivated after jack bean in the period of 70 days of cultivation.

For plant height, there was a significant effect for water sources, with the highest average being for ANE, with 45.51 cm, differing from the height of the beets irrigated with TW, with 37.67 cm.

The leaf area index (LAI) obtained the highest average for the Jack bean treatment and the lowest value for *C. juncea* when irrigated with TW. For plants irrigated with ANE, the highest average was for Jack bean and the lowest for *C. spectabilis* (Table 4). The LAI was 1.29 m^2^ m^−2^ for beets irrigated with TW and 2.62 m^2^ m^−2^ with irrigation by ANE. Tullio et al. (2013) in their studies when evaluating the table beet grown for 65 days found a leaf area index of 1.94 m^2^.

The TSS soluble of the beets did not show significant effects of the treatments, and the average of green fertilizers irrigated with TW was 11.51°Brix, and for ANE, it was 11.57°Brix. Façanha et al. (2010) found mean values of total soluble solids in the tuberous roots of table beet that varied from 10.4°Brix to 11.1°Brix cultivated with bovine manure. Gomes et al. (2015) report SST values of 11.5°Brix in table beet irrigated with anaerobic dairy effluent.

### 3.5 Chemical Composition of Lettuce and Table Beet Leaves

The levels of nitrogen (N), phosphorus (P), and potassium (K) macronutrients were influenced by the interaction among irrigation sources and green manures (Table 8). In TW, the highest N value found was in lettuce in succession to pigeon pea leaves, which was similar to the control, but differed from lettuce in succession to jack bean and *Crotalaria*. Only after *Crotalaria* irrigated with TW did the lettuces have an N content below the reference in the literature, that is, values below 30 g kg^−1^.

**TABLE 8:** Content of macronutrients (g kg^−1^) in lettuce leaves grown in intercropping with table beet, in post‐cultivation with green manures, and irrigated with two sources of water (tap water [TW]; dairy effluent treated by anaerobic system [ANE]).

Treatments	Nitrogen (N)		Phosphorus (P)		Potassium (K)	
	TW	ANE	TW	ANE	TW	ANE
Pigeon pea	44.79Ab	56.00Aa	3.14ABa	3.08ABa	32.83Aa	33.07ABa
Control	36.35ABb	50.93Aa	2.21Cb	2.68Ba	25.74Aa	30.14ABa
Jack bean	31.13BCb	48.74Aa	3.31Aa	3.01ABa	27.61Ab	37.72Aa
*Crotalaria spectabilis*	29.77BCb	53.92Aa	3.24Aa	3.47Aa	29.57Aa	34.29ABa
*Crotalaria juncea*	24.08Cb	51.30Aa	2.68BCb	3.13ABa	29.40Aa	28.92Ba
Mean	33.22	51.78	2.92	3.07	29.03	32.83
Reference^a^	30–50		4–7		50–80	
CV (%)	13.19		7.95		12.30	

Treatments	Calcium (Ca)			Magnesium (Mg)			Sulfur (S)		
	TW	ANE	Mean	TW	ANE	Mean	TW	ANE	Mean
Pigeon pea	9.99	8.71	9.35A	4.52	3.89	4.21AB	2.65	2.49	2.57A
Control	9.84	8.83	9.34A	4.15	3.74	3.94B	2.16	2.39	2.28B
Jack bean	10.81	8.63	9.72A	4.38	3.67	4.02B	2.38	2.49	2.44AB
*C. spectabilis*	9.22	8.76	8.99A	5.02	4.43	4.72A	2.19	2.40	2.29B
*C. juncea*	9.93	8.57	9.25A	4.11	4.04	4.08B	2.09	2.54	2.32B
Mean	9.96a	8.70b		4.44a	3.95b		2.29b	2.46a	
Reference^a^	15–25			4–6			1.5–2.5		
CV (%)	10.06			10.44			8.78		

*Note:* Means followed by different letters, uppercase in the columns and lowercase in the rows, differ by Tukey's test or *F* (*p* < 0.05).

Abbreviation: CV = coefficient of variation.

^a^ Reference values (Raij et al. 1997).

When irrigated with ANE, there was no difference for N among lettuces compared to green manures. However, the N values were higher in lettuce leaves irrigated with ANE compared to TW, which demonstrates that the lettuces absorbed the N supplied by the effluent via irrigation. Urbano et al. (2017) found a value, average of two cycles, of 43.19 g kg^−1^ of N in lettuce cv. Elisa grown with treated sewage effluent.

Lettuce leaf phosphorus (P) contents were below the reference, less than 4 g kg^−1^, with the lowest value being the control lettuce, that is, lettuces benefited from phosphorus recycled by green manures. However, there was no complete synchronization between the need for P for lettuce and the availability of P for green manures.

The levels of potassium (K) and calcium (Ca) in lettuce leaves were below the values recommended by Raij et al. (1997). Urbano et al. (2017) found values of 47.37 and 7.85 g kg^−1^ lettuce cv. Elisa grown with treated sewage effluent, respectively, for K and Ca, that is, also below the concentration described by Raij et al. (1997). Paulus et al. (2012) studied the mineral composition of lettuce cv. Veronica grown in hydroponics with saline waters and found K (48 g kg^−1^) and Ca (10 g kg^−1^) values also below the reference. However, Sandri et al. (2006), studying the cultivation of lettuce cv. Elisa, with effluent composed of domestic and sanitary wastes, found values of K (52.3 g kg^−1^) and Ca (13.2 g kg^−1^), with only calcium slightly below the reference.

The magnesium (Mg) content, on average among TW and ANE, was higher for lettuce, cultivated after global audience, with no difference for pigeon pea and higher than control, beans, and *C. juncea* (Table 9). In the average of green manures, the Mg content of lettuces irrigated with ANE (3.95 g kg^−1^) was lower than that of TW irrigation (4.44 g kg^−1^) (Sandri et al. 2006), selecting a value of 3.0 g kg^−1^, less than that suitable for a lettuce.

**TABLE 9:** Content of macronutrients (g kg^−1^) in table beet leaves cultivated in combination with lettuce, in post‐cultivation with green manures, and irrigated with two sources of water (WS) (tap water [TW]; system treated dairy effluent anaerobic [ANE]).

WS	N	P	K	Ca	Mg	S
TW	35.90b	1.92a	33.04a	14.95a	11.07a	0.99a
ANE	41.88a	1.78b	31.25a	12.82b	11.42a	0.79a
Reference^a^	30–50	2–4	20–40	25–35	3–8	2–4
CV (%)	23.32	10.66	11.04	20.68	15.43	49.88

*Note:* Means followed by different letters, for each parameter, differ by the *F* test (*p* < 0.05).

Abbreviation: CV = coefficient of variation.

^a^ Reference values (Tivelli et al. 2011; Raij et al. 2001).

Sulfur (S) levels, in general, were within the range recommended by Raij et al. (2001). Only for lettuce cultivated in succession to pigeon pea, a higher value (2.57 g kg^−1^) was found, which was above the reference. Sandri et al. (2006) reported values above those suitable for lettuce, ranging from 3.7 to 4.6 g kg^−1^. Paulus et al. (2012) found mean values of 2.1 g kg^−1^.

Regarding the levels of macronutrients in table beet leaves, with the exception of potassium (K), there was no interaction among water sources and green manures. The differences were observed only for water sources (Table 9).

The nitrogen (N) content was higher for beets irrigated with ANE (41.88 g kg^−1^) compared to TW (35.90 g kg^−1^). Gomes et al. (2017) studied the supply of nutrients to table beet cv. Cabernet by irrigation with treated dairy effluents and reported values of 26.82 g kg^−1^, below those found in this experiment. However, the N content of the effluent treated by the anaerobic system was N‐TNK = 42.24 ± 1.77, also below the current experiment (70.92 ± 35.80 mg L^−1^).

The nitrogen (N) content was higher for beets irrigated with ANE (41.88 g kg^−1^) compared to TW (35.90 g kg^−1^). Gomes et al. (2017) studied the supply of nutrients to table beet cv. Cabernet by irrigation with treated dairy effluents and reported values of 26.82 g kg^−1^, below those found in this experiment. However, the N content of the effluent treated by the anaerobic system was N‐TNK = 42.24 ± 1.77, also below the current experiment (70.92 ± 35.80 mg L^−1^).

For phosphorus (P) and calcium (Ca), the highest levels were found in leaves whose plants were irrigated with TW. However, for both nutrients, the values were below the reference values of Raij et al. (2001). The data by Gomes et al. (2017) corroborate the P content, with a value of 1.60 g kg^−1^, when irrigated with anaerobic effluent.

The levels of magnesium (Mg) and sulfur (S) in table beet leaves did not differ between the water sources used for irrigation. Mg was above the recommended range and S was below the reference. Gomes et al. (2017) reported the following values for lettuces irrigated with effluent treated by anaerobic system: K = 25.30 g kg^−1^, Ca = 40.59 g kg^−1^, Mg = 35.77 g kg^−1^, S = 2.95 g kg^−1^.

The content of potassium (K) in the leaves of table beet varied, in the average of the water sources, for the cultivation in succession to green manures: The highest average was verified after *C. juncea* (35.43 g kg^−1^) superior only to control (29.53 g kg^−1^).

**FIGURE 2:**
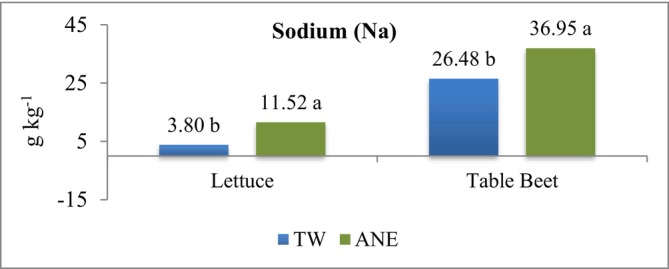
Sodium (Na) content in lettuce and table beet leaves grown in intercropping and irrigated with TW or dairy effluent treated by anaerobic system (ANE). Means followed by different letters, for each culture, differ from each other by the *F* test (*p* < 0.05). CV = 21.15 (lettuce); CV = 31.25 (table beet); CV = coefficient of variation.

The curly lettuce contains 3 mg 100 g^−1^ of sodium (Na), with 96.1% moisture, that is, 0.77 g kg^−1^ of sodium in the dry matter (Taco 2011). The sodium content in the lettuce leaves was not influenced by green manures, only by water sources (Figure 2). Lettuce cultivated with TW irrigation had a content of 3.80 g kg^−1^ and differed from the leaf content of lettuces irrigated with effluent (ANE), which showed 11.52 g kg^−1^. Both values are higher than the reference of the Brazilian Food Composition Table (Taco 2011). Sandri et al. (2006) reported sodium levels in lettuce grown with effluent up to nine times higher than lettuces grown with tap water.

Regarding sodium (Na) in table beet leaves, the highest content occurred when irrigated with ANE (36.95 g kg^−1^), higher than the value found for TW (26.48 g kg^−1^). The Na content in the table beet leaves was higher than the 16.25 g kg^−1^ reported by Tivelli et al. (2011). However, Gomes et al. (2017) reported values of Na = 413.33 g kg^−1^ in table beet leaves irrigated with dairy treated effluent, much higher than those found in the current experiment.

### 3.6 Results of Soil Analysis After Cultivation of Lettuce and Table Beet

The results obtained for electrical conductivity (EC) and pH of the aqueous extract of the soil did not differ among green fertilizers, but in the means among water sources (Table 10), with the highest value found for EC in the treatment with effluent (1.18 dS m^−1^) in relation to TW (0.23 dS m^−1^). Evaluating the aqueous extract in soil saturation paste after cultivation of green manures, it is noted that there was a reduction in the EC of the soil irrigated with TW from 1.56 to 1.18 dS m^−1^. Rossi et al. (2014) observed similar results in their study, in which EC for ANE was superior to water. The total concentration of salts in the irrigation water, without specifying them, is evaluated in relation to the EC, so this explains the higher values in relation to the aqueous extract of soil saturation when subjected to irrigation with effluent.

**TABLE 10:** Electrical conductivity (EC) and pH of the saturation paste of the soil irrigated with tap water (TW) and anaerobic treated dairy effluent (ANE) after the cultivation of green fertilizers and successive cultivation of lettuce and table beet in consortium.

Treatments	Electrical conductivity (EC)	pH
	dS m^−1^	
TW	0.23b	3.99a
ANE	1.18a	4.36b
CV (%)	32.38	12.60

*Note:* Means followed by different letters, for each parameter, differ by the *F* test (*p* < 0.05).

Abbreviation: CV = coefficient of variation.

Regarding the pH of the aqueous extract of saturation of the soil, the values were higher for the treatments that received ANE (4.36) in relation to the TW (3.99). According to results obtained from soil analysis collected after the cultivation of green manures and successive cultivation of the lettuce and table beet mix irrigated with TW or treated dairy effluent (ANE), it was found that there was a difference only among the sources of water (Table 11). When lettuce and table beet in succession intercropped green manure plants were irrigated with ANE, it was observed that, with the exception of potential acidity, the concentrations were higher (K, Ca, and Mg), being that for phosphorus (P) and sulfur (S), there was no statistical difference.

**TABLE 11:** Results of the chemical analysis of the soil after the cultivation of green manures and successive cultivation of the lettuce and table beet intercrop irrigated with tap water (TW) or dairy effluent treated by anaerobic system (ANE).

Treatments	pH (CaCl_2_)	P	S	K	Ca	Mg	Na
		mg dm^−3^		mmolc dm^−3^			
TW	6.48b	21.90a	8.80a	2.13b	61.45b	15.35b	0.43b
ANE	6.56a	21.10a	8.95a	3.11a	65.40a	20.40a	2.10a
CV (%)	1.52	42.54	38.63	20.32	9.61	26.97	47.74

Treatments	OM	H + Al	SB	CEC	V	ESP
	g kg^−1^	mmol_c_ dm^−3^			(%)	(%)
TW	17.88 a	18.07 a	79.35 b	97.55 b	81.19b	0.43b
ANE	17.97 a	16.52 b	91.01 a	107.50 a	84.50a	1.97a
CV (%)	6.34	4.90	11.38	9.24	2.28	50.45

*Note:* Means followed by different letters, for each parameter, differ by the *F* test (*p* < 0.05).

Abbreviation: CV = coefficient of variation.

When lettuce and table beet in succession intercropped green manure plants were irrigated with ANE, it was observed that, with the exception of potential acidity, the concentrations were higher (K, Ca, and Mg), being that for phosphorus (P) and sulfur (S), there was no statistical difference.

The higher levels of macronutrients (potassium [K], calcium [Ca], and magnesium [Mg]) can be explained due to the characteristics of the ANE, which in its physicochemical characterization showed K^+^ = 74.80 ± 50.84 mg L^−1^, Ca^+2^ = 40.89 ± 35.81 mg L^−1^, Mg^+2^ = 69.47 ± 43.49 mg L^−1^, and Na^+^ = 197 ± 101.1 mg L^−1^, thus presenting an effect residual in the soil. Consequently, the sum of bases (SB), base saturation (V%), and the cation exchange capacity (CEC) of these soils showed a significant increase in relation to the soil irrigated with TW. There was an increase in the organic matter content in the soil in relation to green manures, and the OM value of the plots previously cultivated with the *Crotalaria* were higher than the other treatments: In the plots with *C. spectabilis* , the OM value was 19.00 g kg^−1^ and *C. juncea* were 18.23 g kg^−1^, whereas in the control, jack bean and pigeon pea were 17.50, 17.36, and 17.55 g kg^−1^, respectively.

Regarding sodium (Na), a reduction in the residual effect was observed in the soil irrigated with the anaerobic effluent (ANE), with values decreasing from 4.20 mmolc dm^−3^ after the cultivation of green manures to 2.10 mmolc dm^−3^ in the successive cultivation of the lettuce and table beet consortium. Consequently, the ESP also decreased, from 4.39% to 1.97% in the ANE treatment. This reduction indicates a decrease in soil sodicity over the cultivation cycles, suggesting that the combined use of green manures and successive vegetable cultivation favored the desorption and redistribution of Na^+^ in the soil exchange complex.

This behavior may be related to the greater root activity and organic matter input promoted by green manures, which contribute to the increase in cation exchange capacity (CEC) and enhance the retention of Ca^2+^ and Mg^2+^ in exchange sites, displacing Na^+^ to the soil solution where it can be leached by subsequent irrigation. Similar results were reported by Donatti et al. (2017) and Rossi et al. (2014), who observed increases in sum of bases (SB), base saturation (V%), and CEC in soils irrigated with dairy effluent, associated with reductions in exchangeable Na levels.

According to the criteria established by CETESB and ANA (2011), irrigation with wastewater can be safely practiced as long as the ESP remains below 6.0%. Thus, the values obtained in this study—below 2.0% after the successive cultivation—are well within the recommended limits, indicating no risk of sodification under the evaluated conditions. These results reinforce that the agricultural use of treated dairy effluent, when properly managed and associated with green manures, does not lead to sodium accumulation in the soil and may even contribute to the chemical balance and improvement of soil fertility in protected cultivation systems.

## 4 Conclusions

Lettuce was more productive in cultivation in succession to pigeon pea, and the leaves showed the highest levels of nitrogen in this treatment, regardless of the water source. The effluent input positively influenced the production of the table beet roots when in succession of *C. juncea* , jack bean, and pigeon pea. The levels of macronutrients and sodium in table beet leaves were higher when irrigated with dairy effluent treated by anaerobic system.

Irrigation with dairy effluent treated by anaerobic system contributed to the chemical fertility of the soil, increasing macronutrients, sum of bases, v%, and cation exchange capacity.

The intercropping of lettuce and table beet in succession with green manures reduced the levels of NA and consequently the ESP of the soil irrigated with treated dairy effluent from 4.33% to 1.97%.

## Author Contributions

**Juliana de Fátima Vizú:** conceptualization, data curation, formal analysis, investigation, methodology, validation, visualization, writing – original draft, writing – review and editing. **Rogers Ribeiro:** data curation, formal analysis, investigation, methodology, validation, visualization, writing – original draft, writing – review and editing. **Tamara Maria Gomes:** data curation, formal analysis, investigation, methodology, validation, visualization, writing – original draft, writing – review and editing. **Giovana Tommaso:** data curation, formal analysis, investigation, methodology, validation, visualization, writing – original draft, writing – review and editing. **Bruno Fernando Capodifoglio:** data curation, formal analysis, investigation, methodology, validation, visualization, writing – original draft. **Mileni Nobre Cabral:** data curation, formal analysis, investigation, methodology, validation, visualization, writing – original draft. **Ana Claudia Pereira Carvalho:** writing – review and editing. **Fabrício Rossi:** data curation, formal analysis, investigation, methodology, validation, visualization, project administration, supervision, writing – original draft, writing – review and editing.

## Funding

The Article Processing Charge for the publication of this research was funded by the Coordenação de Aperfeiçoamento de Pessoal de Nível Superior ‐ Brasil (CAPES) (ROR identifier: 00x0ma614).

## Conflicts of Interest

The authors declare no conflicts of interest.

## Data Availability

The data that support the findings of this study are available from the corresponding author upon reasonable request.

## References

[wer70221-bib-0001] APHA (American Public Health Association) , AWWA (American Water Works Association) , and WEF (Water Environment Federation) . 2012. “Standard Methods for the Examination for Water and Wastewater.” Washington, 22 ed. 1496p.

[wer70221-bib-0002] Araújo, J. S. , A. P. Andrade , C. I. Ramalho , and C. A. V. Azevedo . 2009. “Características de Frutos de Pimentão Cultivado em Ambiente Protegido Sob Doses de Nitrogênio via Fertirrigação.” Revista Brasileira de Engenharia Agricola e Ambiental 13: 152–157.

[wer70221-bib-0003] Associação Brasileira do Comércio de Sementes e Mudas . 2017. “Mapeamento e Quantificação da Cadeia Produtiva das Hortaliças do Brasil.” Confederação da Agricultura e Pecuária no Brasil – Brasília: CNA, 79p.

[wer70221-bib-0004] Ayers, R. S. , and D. W. Westcot . 1999. “A Qualidade da Água na Agricultura.” In Revisado 1 (FAO). Tradução de Water Quality for Agriculture, edited by H. R. Tradução de Gheyi , J. F. de Meiros , and F. A. V. Damasceno , vol. 29. UFPB.

[wer70221-bib-0005] Bento, T. S. , M. A. C. Carvalho , and W. Gervazio . 2015. “Adubação Verde e Sistemas de Cultivo na Produção Orgânica de Alface.” Cadernos de Agroecologia 9: 1–12.

[wer70221-bib-0058] Cavalcante, V. , et al., 2012. “Biomass and Nutrient Extraction by Cover Crops.” Revista Brasileira de Engenharia Agrícola e Ambiental 16: 521–528.

[wer70221-bib-0006] CETESB/ANA (Companhia de Tecnologia de Saneamento Ambiental/Agencia Nacional de Águas) . 2011. In Guia Nacional de Coleta e Preservação de Amostras: Água, Sedimento, Comunidades Aquáticas e Efluentes Líquidos, edited by C. J. Brandão et al. ANA.

[wer70221-bib-0055] CETESB ‐ Environmental Sanitation Technology Company . 2006. Guidelines for the Preparation of Projects Aimed at the Application of Reuse Water From Domestic Wastewater Treatment Plants in Agriculture, 11 p. CETESB.

[wer70221-bib-0007] Chadwick, M. , J. R. Swann , F. Gawthrop , et al. 2024. “Mapping Taste and Flavour Traits to Genetic Markers in Lettuce *Lactuca sativa* .” Food Chemistry: Molecular Sciences 9: 100215. 10.1016/j.fochms.2024.100215. 39281292 PMC11399806

[wer70221-bib-0008] Donatti, R. N. , T. M. Gomes , L. C. Menegassi , G. Tommaso , and F. Rossi . 2017. “Sodium Phytoremediation by Green Manure Growing in Soil Irrigated With Wastewater of Dairy Industry.” Engenharia Agrícola v.37: 665–675.

[wer70221-bib-0009] Dong, S. , G. Gai , Y. Shi , et al. 2024. “Partial Substitution of Chemical Fertilizer by Green Manure Increases Succeeding Maize Yield and Annual Economic Benefit in Low‐Yield Cropland in the Yellow River Delta.” Soil Use and Management 40, no. 1: e13022. 10.1111/sum.13022.

[wer70221-bib-0057] dos Santos, A. N. , Ê. F. de França e Silva , G. F. da Silva , R. R. Bezerra , and E. M. R. Pedrosa . 2017. “Nutrient Concentration in Cherry Tomato Under Management Strategies for Applying Nutrient Solution With Brackish Water.” Revista Ciência Agronômica 48: 576–585.

[wer70221-bib-0011] dos Santos, H. G. , et al., 2013. Sistema Brasileiro de Classificação de Solos, 353 p., 3rd ed. rev. e ampl. Empresa Brasileira de Pesquisa Agropecuária (Embrapa).

[wer70221-bib-0012] Façanha, M. L. , D. C. Medeiros , O. L. Coutinho , L. F. Marques , C. B. Medeiros , and L. S. Vale . 2010. “Produção e Qualidade da Beterraba em Função da Adubação com Esterco Bovino.” Revista Brasileira de Agroecologia 5: 24–31.

[wer70221-bib-0013] FAO (Food and Agriculture Organization of the United Nations) . 2008. “Land Resources, Management, Planning and Use.” Disponível em: http://www.fao.org/ag/agl/agll/spush. Acesso em: 12 fev. 2018.

[wer70221-bib-0014] Farias, J. R. B. , H. Bergamaschi , and S. R. Martins . 1994. “Evapotranspiração no Interior de Estufas Plásticas.” Revista Brasileira de Agrometeorologia 2: 17–22.

[wer70221-bib-0015] Ferreira, D. F. 2011. “Sisvar: A Computer Statistical Analysis System.” Ciência e Agrotecnologia 35: 1039–1042.

[wer70221-bib-0056] Garcia, G. O. , P. A. Ferreira , G. V. Miranda , J. C. L. Neves , W. B. Moraes , and D. B. dos Santos . 2007. “Leaf Contents of Cationic Macronutrients and Their Relationships With Sodium in Maize Plants Under Salt Stress.” Idesia 25: 93–106.

[wer70221-bib-0016] Gomes, T. M. , F. Rossi , G. Tommaso , R. Ribeiro , M. M. Kushida , and M. J. Stablein . 2017. “Supplementation of Nutrients for Table Beets by Irrigation With Treated Dairy Effluent.” Engenharia Agrícola 37: 1137–1147.

[wer70221-bib-0017] Gomes, T. M. , F. Rossi , G. Tommaso , R. Ribeiro , N. P. F. Macan , and R. S. Pereira . 2015. “Treated Dairy Wastewater Effect on the Yield and Quality of Drip Irrigated Table Beet.” Applied Engineering in Agriculture 31: 255–260.

[wer70221-bib-0018] Humphries, A. W. , R. A. Latta , G. C. Auricht , and W. D. Bellotti . 2004. “Over‐Cropping Lucerne With Wheat: Effect of Lucerne Winter Activity on Total Plant Production and Water Use of the Mixture, and Wheat Yield and Quality.” Australian Journal of Agricultural Research 55: 839–848.

[wer70221-bib-0019] Juchen, C. R. , F. L. Suszek , and M. A. Vilas Boas . 2013. “Irrigação por Gotejamento Para Produção de Alface Fertirrigada com Águas Residuárias Agroindustriais.” Irriga 18: 243–256.

[wer70221-bib-0020] Kama, R. , J. X. He , F. Nabi , et al. 2025. “Crop Rotation and Green Manure Type Enhance Organic Carbon Fractions and Reduce Soil Arsenic Content.” Agriculture, Ecosystems and Environment 378: 109287. 10.1016/j.agee.2024.109287.

[wer70221-bib-0021] Kathiravan, G. , J. Churaman , and N. Felix . 2024. “Assessing Sensory Attributes and Quality of Lettuce From Open Field, Greenhouse, and Controlled Environment Production Systems.” Food and Humanity 2: 100311. 10.1016/j.foohum.2024.100311.

[wer70221-bib-0022] Kist, B. B. , and R. R. Beling . 2023. Anuário Brasileiro de Horti&fruti. Editora Gazeta Santa Cruz.

[wer70221-bib-0023] Köppen, W. , and R. Geiger . 1928. Klimate der Erde. Verlag Justus Perthes.

[wer70221-bib-0024] Magalhães, F. F. , F. F. Cunha , A. R. Godoy , E. J. Souza , and T. R. Silva . 2015. “Produção de Cultivares de Alface Tipo Crespa sob Diferentes Lâminas de Irrigação.” Water Resources and Irrigation Management 4: 41–50.

[wer70221-bib-0025] Malavolta, E. , G. C. Vitti , and S. A. Oliveira . 1997. Avaliação do Estado Nutricional das Plantas: Princípios e Aplicações. 2nd ed. Potafos.

[wer70221-bib-0026] Mishra, S. , R. Kumar , and M. Kumar . 2023. “Use of Treated Sewage or Wastewater as an Irrigation Water for Agricultural Purposes‐Environmental, Health, and Economic Impacts.” Total Environment Research Themes 6: 100051. 10.1016/j.totert.2023.100051.

[wer70221-bib-0027] Oliveira, F. F. , J. G. M. Guerra , D. L. Almeida , et al. 2008. “Avaliação de Coberturas Mortas em Cultura de Alface sob Manejo Orgânico.” Horticultura Brasileira 26: 216–220.

[wer70221-bib-0028] Pauletti, V. , A. C. V. Motta , B. M. Serrat , N. Favaretto , and A. dos Anjos . 2009. “Atributos Químicos de um Latossolo Bruno sob Sistema Plantio Direto em Função da Estratégia de Adubação e do Método de Amostragem de Solo.” Revista Brasileira de Ciência do Solo 33: 581–590.

[wer70221-bib-0029] Paulus, D. , E. Paulus , G. A. Nava , and C. A. Moura . 2012. “Crescimento, Consumo Hídrico e Composição Mineral de Alface Cultivada em Hidroponia com Águas Salinas.” Revista Ceres 59, no. 1: 110–117.

[wer70221-bib-0030] Pedrero, F. , and J. J. Alarcón . 2009. “Effects of Treated Wastewater Irrigation on Lemon Trees.” Desalination 246: 631–639.

[wer70221-bib-0059] Pereira, G. A. M. , et al., 2012. “Phytomass of Green Manures and Soil Cover in the Alto Vale do Jequitinhonha Region, Minas Gerais.” Revista Agro@mbiente On‐line 6, no. 2: 110–116.

[wer70221-bib-0031] Persiani, A. , M. Diacono , and F. Montemurro . 2024. “The Impact of Long‐Term Organic Horticultural Systems on Energy Outputs and Carbon Storages in Relation to Extreme Rainfall Events.” European Journal of Agronomy 161: e127398. 10.1016/j.eja.2024.127398.

[wer70221-bib-0061] Purquerio, L. F. V. , et al., 2011. “Lettuce Production in a Greenhouse With Soil Salinized After the Cultivation of Nutrient‐Extracting Plants.” Horticultura Brasileira 29.

[wer70221-bib-0032] Qing, G. , S. L. Foster , Z. Anari , M. Matlock , G. Thoma , and L. F. Greenlee . 2021. “Disinfection/Ammonia Removal From Aquaculture Wastewater and Disinfection of Irrigation Water Using Electrochemical Flow Cells: A Case Study in Hawaii.” Water Environment Research 93, no. 10: 2149–2168. 10.1002/wer.1588. 34022089

[wer70221-bib-0033] Raij, B. , H. Cantarella , J. Á. Quaggio , and A. M. C. Furlani . 1997. “Recomendações de adubação e Calagem Para o Estado de São Paulo.” Instituto Agronômico/Fundação IAC, (Boletim Técnico, 100).

[wer70221-bib-0034] Raij, B. V. , J. C. Andrade , H. Cantarella , and J. A. Quaggio . 2001. Análise Química Para Avaliação da Fertilidade de Solos Tropicais. Instituto Agronômico.

[wer70221-bib-0035] Ramírez‐Pedraza, A. , S. Salazar‐Colores , J. Terven , J. Á. Romero‐González , J. J. González‐Barbosa , and D. M. Córdova‐Esparza . 2024. “Nutritional Monitoring of Rhodena Lettuce via Neural Networks and Point Cloud Analysis.” AgriEngineering 6, no. 3: 3474–3493. 10.3390/agriengineering6030198.

[wer70221-bib-0036] Richards, L. A. 1954. Diagnosis and Improvement of Saline and Alkali Soils. Vol. 60. United States Salinity Laboratory, USDA, Agriculture Handbook.

[wer70221-bib-0037] Rossi, F. , T. Gomes , J. C. H. B. Tol , M. R. Ferraz , P. H. C. Luz , and E. J. Ambrosano . 2014. “Fitoextração de Sódio Pelo Cultivo do Milheto em Sucessão a Produção da Beterraba Irrigada com Águas Residuárias de Origem Agroindustrial.” Cadernos de Agroecologia 4: 1–11.

[wer70221-bib-0038] Sandri, D. , E. E. Matsura , and R. Testezlaf . 2006. “Teores de Nutrientes na Alface Irrigada com Água Residuária Aplicada por Sistemas de Irrigação.” Engenharia Agrícola 26: 45–57.

[wer70221-bib-0039] Sandri, D. , E. E. Matsura , and R. Testezlaf . 2007. “Desenvolvimento da Alface Elisa em Diferentes Sistemas de Irrigação com Água Residuária.” Revista Brasileira de Engenharia Agricola e Ambiental v.11: 17–29.

[wer70221-bib-0040] Sarmento, A. L. R. , F. H. F. Pereira , M. C. Silva , J. E. de Medeiros , and E. C. B. S. Freire . 2011. “Fontes e Tempo de Incorporação de Estercos no Cultivo da Beterraba.” Revista Verde 6: 50–58.

[wer70221-bib-0041] Sediyama, M. A. N. , M. R. Santos , S. M. Vidigal , and L. T. Salgado . 2011. “Produtividade e Exportação de Nutrientes em Beterraba Cultivada com Cobertura Morta e Adubação Orgânica.” Revista Brasileira de Engenharia Agricola e Ambiental 15, no. 9: 883–889.

[wer70221-bib-0042] Sediyama, M. A. N. , M. R. Santos , S. M. Vidigal , I. C. Santos , and L. T. Salgado . 2010. “Ocorrência de Plantas Daninhas no Cultivo de Beterraba com Cobertura Morta e Adubação Orgânica.” Planta Daninha 28, no. 4: 717–725.

[wer70221-bib-0043] Shahrivar, A. A. , D. Hagare , B. Maheshwari , and M. M. Rahman . 2023. “The Impact of Irrigation With Treated Wastewaters on Soil and Kikuyu Grass Nutrient Compositions.” Water Environment Research 95, no. 6: e10873. 10.1002/wer.10873. 37218371

[wer70221-bib-0044] Shi, M. , J. Gu , H. Wu , et al. 2022. “Phytochemicals, Nutrition, Metabolism, Bioavailability, and Health Benefits in Lettuce—A Comprehensive Review.” Antioxidants 11, no. 6: 1158. 10.3390/antiox11061158. 35740055 PMC9219965

[wer70221-bib-0045] Silva, A. O. , E. F. F. Silva , and A. E. Klar . 2013. “Eficiência de Uso da Água em Cultivares de Beterraba Submetidas a Diferentes Tensões da Água no Solo.” Water Resources and Irrigation Management 2: 27–36.

[wer70221-bib-0046] Silva, R. C. , F. F. Oliveira , K. R. Souza , E. S. Brito , A. O. Silva , and C. M. L. Guedes . 2017. “Avaliação de Diferentes Coberturas Mortas na Produção de Beterraba (*Beta vulgaris* L.).” Revista Semiárido De Visu 5: 3–10.

[wer70221-bib-0047] Siwek, P. , P. Bucki , I. Domagała‐Świątkiewicz , and P. Lalewicz . 2024. “Effect of Cover Crops Integration in Crop Rotation on the Yield and Chemical Composition of Edible Parts of Vegetables Grown in an Organic System in High Tunnel.” Scientia Horticulturae 332: 113191. 10.1016/j.scienta.2024.113191.

[wer70221-bib-0060] Soares, C. M. J. , et al., 2015. “Production of Green Manures in the Cerrado and Their Effects on Weeds.” Revista de Ciências Agroambientais 13: 57–64.

[wer70221-bib-0048] Taco ‐ Tabela Brasileira de Composição de Alimentos . 2011. “Campinas: NEPA‐UNICAMP, 4^a^ edição, 161 p”.

[wer70221-bib-0049] Taiz, L. , and E. Zeiger . 2013. Fisiologia Vegetal. 5th ed. Artmed.

[wer70221-bib-0050] Taylor, R. P. , C. L. W. Jones , M. Laing , and J. Dames . 2018. “The Potential Use of Treated Brewery Effluent as a Water and Nutrient Source in Irrigated Crop Production.” Water Resources and Industry 19: 47–60.

[wer70221-bib-0051] Tivelli, S. W. , T. L. Factor , J. R. S. Teramoto , et al. 2011. “Beterraba: Do Plantio à Comercialização.” Boletim Técnico IAC, 210. Instituto Agronômico, 45.

[wer70221-bib-0052] Trani, P. E. , L. F. V. Purqueiro , G. J. B. Figueiredo , S. F. Blat , and C. P. Costa . 2014. “Alface – *Lactuca sativa* L.” In Instruções Agrícolas Para as Principais Culturas Econômicas. Instituto Agronômico.

[wer70221-bib-0053] Tullio, J. A. , R. F. Otto , A. Boer , and O.H.S.E., S . 2013. “Cultivo de Beterraba em Ambientes Protegido e Natural na Época de Verão.” Revista Brasileira de Engenharia Agricola e Ambiental 17: 1074–1079.

[wer70221-bib-0054] Urbano, V. R. , T. G. Mendonça , R. G. Bastos , and C. F. Souza . 2017. “Effects of Treated Wastewater Irrigation on Soil Properties and Lettuce Yield.” Agricultural Water Management 181: 108–115.

